# Efficient *E. coli* Expression Strategies for Production of Soluble Human Crystallin ALDH3A1

**DOI:** 10.1371/journal.pone.0056582

**Published:** 2013-02-22

**Authors:** Georgia-Persephoni Voulgaridou, Theodora Mantso, Katerina Chlichlia, Mihalis I. Panayiotidis, Aglaia Pappa

**Affiliations:** 1 Department of Molecular Biology and Genetics, Democritus University of Thrace, University Campus, Dragana, Alexandroupolis, Greece; 2 Laboratory of Pathological Anatomy, Medical School, University of Ioannina, University Campus, Ioannina, Greece; National Centre for Cell Science, India

## Abstract

Aldehyde dehydrogenase 3A1 (ALDH3A1) is a recently characterized corneal crystallin with its exact functions still being unclear. Expressing recombinant human ALDH3A1 has been difficult in *Escherichia coli (E. coli)* because of low solubility, yield and insufficient purity issues. In this report, we compared different *E. coli* expression strategies (namely the maltose binding protein; MBP- and the 6-his-tagged expression systems) under conditions of auto-induction and co-expression with *E. coli’s* molecular chaperones where appropriate. Thus, we aimed to screen the efficiency of these expression strategies in order to improve solubility of recombinant ALDH3A1 when expressed in *E. coli*. We showed that the MBP- tagged expression in combination with lower-temperature culture conditions resulted in active soluble recombinant ALDH3A1. Expression of the fused 6-his tagged-ALDH3A1 protein resulted in poor solubility and neither lowering temperature culture conditions nor the auto-induction strategy improved its solubility. Furthermore, higher yield of soluble, active native form of 6-his tagged-ALDH3A1 was facilitated through co-expression of the two groups of *E. coli*’s molecular chaperones, GroES/GroEL and DnaK/DnaJ/GrpE. Convenient one step immobilized affinity chromatography methods were utilized to purify the fused ALDH3A1 hybrids. Both fusion proteins retained their biological activity and could be used directly without removing the fusion tags. Taken together, our results provide a rational option for producing sufficient amounts of soluble and active recombinant ALDH3A1 using the *E. coli* expression system for conducting functional studies towards elucidating the biological role(s) of this interesting corneal crystallin.

## Introduction

Human crystallin ALDH3A1 is an NADP^(+)^-dependent enzyme existing as a dimer of 54 kDa subunits. The protein is contained in high amounts in the cornea of mammals ranging between 5–50% of the total water-soluble protein content (depending on species) but is almost absent in the cornea of other species [1]–[3]. Similar to other catalytically active enzymes recruited as taxon-specific corneal crystallins [4], ALDH3A1 is a metabolic enzyme catalyzing the oxidation of aldehydes to their corresponding acids demonstrating high substrate specificity for medium-chain saturated and un-saturated aldehydes [5]. Its metabolic activity appears to be related with the protein’s protective role in corneal epithelium against oxidative damage caused by aldehyde by-products of lipid peroxidation under conditions of cellular stress, e.g. UV-induced oxidative stress [6], [7]. However, constitutive expression of ALDH3A1 in the mammalian cornea exceeds, by far, the levels required for a pure metabolic function. Thus, other potential roles are being explored including: (i) generation of the antioxidant NADPH [8], (ii) direct absorption of UV-energy [9], (iii) scavenging of reactive oxygen species (ROS) [10] and (iv) possession of chaperone-like activity [3], suggesting that ALDH3A1 may contribute to the optical properties of the cornea as well [1], [3]. Furthermore, its absence has been linked with cataract phenotype and ocular oxidative damage in ALDH3A1-null mice [11], whereas recent studies implicate its involvement in cell homeostatic pathways, such as apoptosis, cell cycle regulation, proteasome degradation and DNA damage response [6], [7], [11], [12].

The purpose of the present study was to employ *E. coli* recombinant methods in order to produce substantial amounts of human ALDH3A1 with the aim to direct future studies towards elucidating the biological functions of ALDH3A1. To this end, *E. coli* is the preferred organism for heterologous protein expression due to its many advantages including: (i) the ability to grow quickly into high cell densities, (ii) the requirement of non expensive carbon sources and (iii) its extensively studied physiology [13], [14]. In addition, the wider range of commercial products available for all steps of expression and purification using *E. coli* makes this system even more operable. However, miss-folding and aggregation of recombinant proteins within inclusion bodies in bacteria hinders the successful production of many eukaryotic proteins [13], [15]–[17]. Furthermore, on the occurrence of insolubility issues, both the type of fusion tag and the purification method to be used, constitute critical parameters. In addition, although known tags like maltose binding protein (MBP), can contribute to greater protein solubility [18], [19], culture temperature conditions (during induction) also appear to be a detrimental factor in the production of native protein [20]. Furthermore, methods like auto-induction can be used for easier handling of cultures in combination with high protein yield [21] whereas co-expression of certain bacterial molecular chaperones can assist in the conformational process of the native protein [22].

In the present study, we report the expression of soluble MBP-fused and his-tagged recombinant human crystallin ALDH3A1 in substantial amounts, in *E. coli*, and their purification to homogeneity.

## Materials and Methods

### Materials

Vectors pMAL-c2X and pET-26b(+) were purchased by New England Biolabs (Beverly, MA, USA) and Novagen (EMD Millipore Corporation, Billerica, MA, USA) respectively. All primers were obtained by Invitrogen (Carlsband, CA, USA) while the restriction enzymes and the chaperone plasmid set were from Takara (Shiga, Japan). *Pfu* polymerase and the DNA ligase kit were purchased from Fermentas (Burlington, ON, Canada). Amylose resin was purchased by New England Biolabs (Beverly, MA, USA), while Ni-NTA resin by Qiagen (Venlo, Netherlands). Medium for bacterial cultures along with antibiotics and inducers were purchased either from Applichem (Darmstadt, Germany) or from Sigma-Aldrich Co. (Taufkirchen, Germany). Protease inhibitors as well as the chemicals for the ALDH3A1 enzymatic activity were obtained by Sigma-Aldrich Co. For western blotting, PVDF membranes were purchased from Millipore (Bedford, MA, USA), whereas the chemiluminescence reagents were from Thermo Scientific (Rockford, IL, USA) and the autoradiography films from Genesee Scientific (San Diego, CA, USA). Rabbit polyclonal antibody against human ALDH3A1 was obtained from Abgent (San Diego, CA, USA) and the goat anti-rabbit IgG horseradish peroxidise conjugated antibody was purchased by Millipore (Bedford, MA, USA).

### pMAL/ALDH3A1 Vector Construction

**Figure 1:**
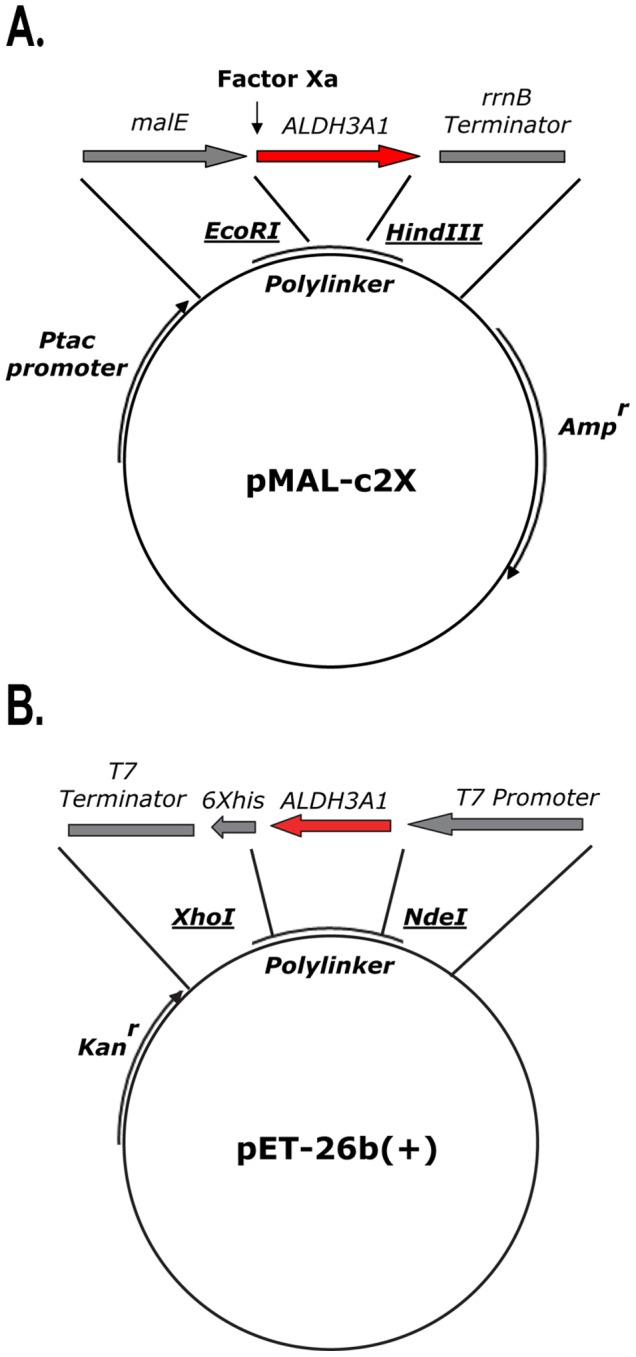
Cloning of the coding sequence of ALDH3A1 into the expression vectors. (A) Construction of the pMAL/hALDH3A1 vector. The HindIII/EcoRI fragment of the PCR product containing the coding region of human ALDH3A1 was inserted into the HindIII and EcoRI sites of the pMAL vector. (B) Construction of the pET-26b (+)/hALDH3A1 vector. The NdeI/XhoI fragment of the PCR product containing the coding region of human ALDH3A1 was inserted into the NdeI and XhoI sites of the pET-26b(+) vector.

To construct the pMAL/ALDH3A1 expression vector, the ΔpCEP4Δ/ALDH3A1 plasmid (containing a full-length of human ALDH3A1 cDNA) was used as the template for the polymerase chain reaction [6], [23]. The following two primers were synthesized in order to: (i) amplify the entire coding sequence and (ii) introduce an *EcoRI* restriction site at the 5′ end and a *HindIII* site on the 3′ end: 1.forward primer: 5′-CT**GAATTC**AGCAAGATCAGCGAG-3′ and, 2.reverse primer: 5′-CT**AAGCTT**TCAGTGCTGGGTCAT-3′. The PCR conditions for the amplification were: 94°C for 60 sec and further 94°C for 30 sec, 60°C for 60 sec, 72°C for 90 sec (for 30 cycles) and a final step at 72°C for 10 min. The *EcoRI HindIII* fragment of the PCR product was inserted into the *EcoRI* and *HindIII* sites of the pMAL expression vector. The resulting vector, pMAL/ALDH3A1 (Figure 1A) was verified by restriction digestion and sequencing from both ends of the inserted fragment.

### ALDH3A1-MBP Tagged Expression

For the expression of ALDH3A1 protein tagged with MBP, BL21(-) *E.coli* cells were transformed with the pMAL/ALDH3A1 construct and incubated in LB broth (with 0.2% glucose and 100 µg/ml ampicillin for selection). The medium was inoculated with an overnight culture (1∶100 dilution) and the culture was incubated under shaking at 37°C until an OD_600_ = ∼ 0.5 was reached. Subsequently, 0.5 mM IPTG was added and the culture was further incubated at 37°C or 25°C for 6 hours.

### pET-26b(+)/hALDH3A1 Vector Construction

The coding sequence of human ALDH3A1 was amplified once more from the ΔpCEP4Δ/hALDH3A1 plasmid by PCR. The following two primers were designed so as to introduce an *NdeI* restriction site at the 5′ end and an *XhoI* restriction site at the 3′ end of the ALDH3A1 coding sequence: 1.forward primer: 5′-GGGAATTC**CATATG**AGCAAGATCAGCGAG-3′ and, 2.reverse primer: 5′-CCG**CTCGAG**GTGCTGGGTCAT-3′. The PCR conditions used for amplification were: initial denaturation at 95°C (90 sec), denaturation at 95°C (30 sec), annealing at 56°C (90 sec), extension at 72°C (120 sec) for 30 cycles and an extra step of 10 min incubation at 72°C. The *NdeI/XhoI* fragment of the PCR product was inserted into the *NdeI* and *XhoI* sites of the of pET-26b(+) expression vector. The resulting vector of pET-26b(+)/ALDH3A1 (Figure 1B) was verified by restriction digestion and sequencing from both ends of the inserted fragment.

### ALDH3A1 his-tagged Expression

BL21(DE3) *E.coli* transformed with pET-26b(+)/ALDH3A1 were cultured at 37°C, in the presence of 30 µg/ml kanamycin for plasmid selection, and when OD_600_ reached ∼ 0.5, 0.5 mM IPTG was added and incubation continued at 18–37°C for 6 hours.

### Auto-induction

For auto-induction, ZYM-5052 medium was used containing 1% tryptone, 0.5% yeast extract, 25 mM Na_2_HPO_4_, 25 mM KH_2_PO_4_, 50 mM NH_4_Cl, 5 mM Na_2_SO_4_, 2mM MgSO_4_, 0.5% glycerol, 0.05% glucose and 0.2% α-lactose as previously described [21]. An overnight culture of BL21(DE3) transformed with pET-26b(+)/ALDH3A1 was used for inoculation at a dilution of 1∶1000. Cultures were incubated (in ZYM-5052 medium with 100 µg/ml kanamycin) at 18°C, 25°C and 37°C, for 9 hours.

### Molecular Chaperone Co-expression

The pET-26b(+)/ALDH3A1 transformed BL21(DE3) *E.coli* were re-transformed with one of the set’s plasmids: pG-KJE8, pGro7, pKJE7, pG-Tf2 and pTf16 and cultured in LB broth with 20 µg/ml chloramphenicol, along with 30 µg/ml kanamycin for the selection of the transformed clones. For protein expression, cells were incubated in shaking cultures at 37°C and in the presence of the appropriate chaperone inducer (0.5 mg/ml L-arabinose and/or 5 ng/ml tetracycline) for allowing the chaperones to be already expressed at the time of ALDH3A1 induction (Table 1). When culture reached OD_600_ ∼ 0.6, 0.5mM IPTG was added and the incubation proceeded for 6 hours at 25°C.

**Table 1:** Description of the chaperone plasmids used in the study.

Plasmid	Resistance Marker	Inducer	Chaperones	Molecular Weights
**pG-KJE8**	Chloramphenicol (20 µg/ml)	L-arabinose (0.5 mg/ml) tetracycline (5 ng/ml)	dnaK-dnaJ-grpE/groES-groEL	dnaK-70 kDa dnaJ-40 kDa grpE-22 kDa groES-10 kDa groEL-60 kDa
**pGro7**	Chloramphenicol (20 µg/ml)	L-arabinose (0.5 mg/ml)	groES-groEL	groES-10 kDa groEL-60 kDa
**pKJE7**	Chloramphenicol (20 µg/ml)	L-arabinose (0.5 mg/ml)	dnaK-dnaJ-grpE	dnaK-70 kDa dnaJ-40 kDa grpE-22 kDa
**pG-Tf2**	Chloramphenicol (20 µg/ml)	tetracycline (5 ng/ml)	groES-groEL/tig	groES-10kDa groEL-60 kDa tig-56 kDa
**pTf16**	Chloramphenicol (20 µg/ml)	L-arabinose (0.5 mg/ml)	tig	tig-56 kDa

### ALDH3A1 Enzymatic Activity

ALDH3A1 activity determinations were carried out using a spectrophotometer (Libra S22, Biochrom Ltd, Cambridge, UK) by monitoring NADPH production at 340 nm as described previously [6]. Briefly, a total of 1 ml reaction containing sodium pyrophosphate (100 mM, pH 8.0), 1 mM pyrazole and 2.5 mM NADP^+^ (co-enzyme) and recombinant ALDH3A1 at various concentrations were prepared and incubated at 25°C. The reaction was initiated using benzaldehyde as a substrate (to a final concentration of 0.5 mM) and was monitored as an increase in NADPH at 340 nm for 5 min. Enzyme activity was calculated using a molar extinction coefficient of 6.22 mM^−1^/cm^−1^ for NADPH. Enzyme specific activities are expressed as nmoles of NADPH/min/mg protein.

### Purification of the ALDH3A1/MBP Recombinant Protein

Cells were harvested through centrifugation at 4.000xg, at 4°C for 20 min and lysed in 20 mM Tris-HCl pH 7.4, 200 mM NaCl and 1 mM EDTA (with the addition of the protease inhibitors: 100 µg/ml PMSF, 0.5 µg/ml leupeptin, 0.5 µg/ml aprotinin and 1 µg/ml pepsatin) by sonication for 8s using the UP50H sonifier by Hielscher Ultrasonics GmbH (Teltow, Germany) at an intermediate setting (cycle 1, 70% amplitude). The lysates were cooled on ice for 30s and the procedure was repeated for a total of 6 cycles. Crude extract was isolated by centrifugation of samples at 9.000x*g* (4°C) for 30 min and further applied to an amylose resin column, already equilibrated with the lysis buffer. Recombinant protein was eluted after the addition of lysis buffer with 10 mM maltose. The presence of ALDH3A1 throughout *E. coli* expression and purification steps was determined by SDS-PAGE and western blot analysis.

### Purification of the ALDH3A1/6xHis Recombinant Protein

Cells were collected and placed in lysis buffer (50 mM NaH_2_PO_4_, 300 mM NaCl, 1% Tween-20, 20 mM imidazole, pH 8.0) in the presence of protease inhibitors as mentioned above. Purification was conducted *via* affinity chromatography, by Ni*-*NTA resin. For the two washes, the concentration of imidazole in the buffer was increased to 40 and 70mM respectively. Bounded protein was eluted through the addition of elution buffer (50 mM NaH_2_PO_4_, 300 mM NaCl, 300 mM imidazole, pH 8.0).

### Western Blotting

Elution parts of ALDH3A1 purification were subjected to SDS-PAGE electrophoresis. The separated proteins were then transferred to a PVDF membrane (Polyvinylidene), which was subsequently blocked with 5% of non-fat dry milk in TBST solution (100 mM Tris, 150 mM NaCl, 0.1% Tween-20). Membrane was subsequently incubated overnight (at 4°C) with the primary, polyclonal anti-ALDH3A1 antibody at a dilution of 1∶500 in 5% non-fat dry milk in TBST, while the secondary horseradish peroxidase conjugated goat anti-rabbit IgG was used in a dilution of 1∶5000 (1 hour incubation). Finally, the protein bands were visualized by the SuperSignal West Pico Chemiluminescent Substrate (Thermo Scientific) for alkaline phosphatase-conjugated secondary antibody as described by the manufacturer.

## Results

### Expression of MBP Fused ALDH3A1 Leads to High Yield and Sufficient Solubility

**Figure 2:**
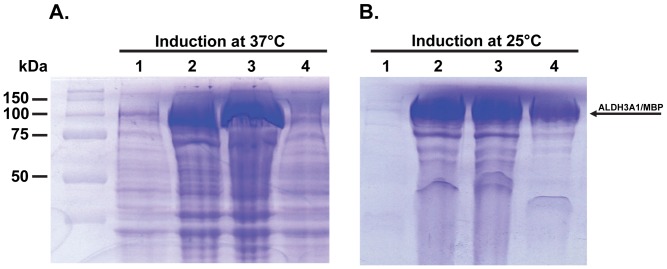
ALDH3A1 heterologous expression through the pMAL-c2X expression system. SDS-PAGE pattern showing induction of ALDH3A1 expression at (A) 37°C and (B) 25°C. Samples were subjected to SDS-PAGE and stained with Coomassie blue. Lane 1, whole cell lysate prior IPTG induction; Lane 2, whole cell lysate 6 hours after IPTG induction; Lane 3, insoluble fraction; Lane 4: soluble fraction, respectively, 6 hours after IPTG induction. The arrow indicates the position of ALDH3A1/MBP protein.

Initially, we tested the expression of the MBP tagged ALDH3A1, through the pMAL-c2X system, given that MBP is known to contribute to increased solubility of the heterologous produced proteins. As it is shown (Figure 2A), ALDH3A1 was expressed at high yields, but with extremely low solubility, when the induction occurred at 37°C (Table 2). After lowering cultivation temperature to 25°C, during induction time (a common strategy to overcome insolubility), significant improvement in ALDH3A1 solubility was observed by increasing from 2.5% at 37°C to 35.5% at 25°C (Figure 2B; Table 2). The recombinant MBP-fused ALDH3A1 was found to be functionally active when tested for the presence of ALDH3A1 enzymatic activity even though it was tagged with the 42kDa MBP (Table 3, specific activity of the crude extract).

**Table 2:** Comparison of different *E.coli* strategies for production of soluble recombinant ALDH3A1.

Method of expression	Induction temperature (°C )	Solubility (% of the induced protein)^a^
ALDH3A1/MBP tagged	37	2.5±0.59
	25	35.47±3.29
ALDH3A1/6xHis tagged	37	2.02±0.57
	25	3.45±1.26
	18	2.73±0.48
ALDH3A1/6xHis tagged (autoinduction)	37	3.11±0.34
	25	4.02±0.11
	18	2.96±0.59
ALDH3A1/6xHis tagged **pG-KJE8**	25	19.54±1.01
ALDH3A1/6xHis tagged **pGro7**	25	11.63±1.03
ALDH3A1/6xHis tagged **pKJE7**	25	4.36±0.36
ALDH3A1/6xHis tagged **pG-TF2**	25	3.75±0.48
ALDH3A1/6xHis tagged **pTF16**	25	6.29±1.01

a The % solubility values reported are mean of three different experiments.

**Table 3:** Purification of MBP-tagged recombinant human ALDH3A1 from *E. coli*.

Purification Steps	Total Protein (mg)	Yield (%)	Specific Activity^b^ (mU/mg protein)	Purification (fold)
Crude supernatant^a^	62.5	100	90	1
Amylose resin column	3.2	5.12	250	2.77

a The starting material was 250 ml of crude *E. coli* supernatant.

b One milliunit (mU) of activity was defined as the amount of activity that oxidized of 1 nmol of NADPH/min at 25°C. Representative results of three different isolation procedures.

### Expression of his-tagged ALDH3A1 Leads to High Protein Yield but Insufficient Solubility

**Figure 3:**
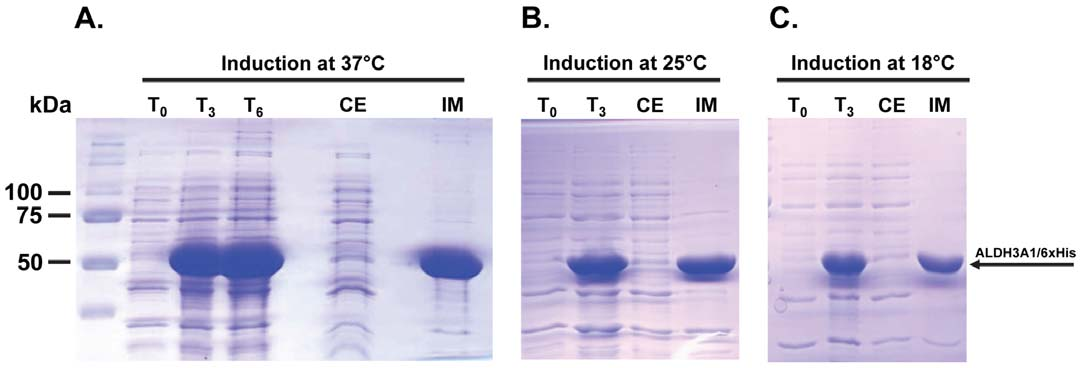
ALDH3A1 heterologous expression through the pET-26b(+) expression system. Induction at (A) 37°C, (B) 25°C and (C) 18°C. Samples were subjected to SDS-PAGE and stained with Coomassie blue. T_0_: Total cell extract form bacterial culture prior of protein induction, T_3_: Total cell extract 3 hours after induction, T_6_: Total cell extract 6 hours after induction, CE: Crude extract of lysed cells 6 hours after induction, IM: Insoluble matter of lysed cells 6 hours after induction. The arrow indicates the position of ALDH3A1/6xHis protein.

Although the MBP tag facilitated the expression of the target heterologous protein, there are further limitations considering the usage of the MBP-fused recombinant proteins including the fact that a number of assays do not allow for the use of a tag with the size of MBP. To this end, the usage of the specialized protease, factor Xa, could solve the problem but the required extra step of the purification process could be a limitation. Consequently, we sought to examine the expression of recombinant human ALDH3A1 fused with a rather smaller but very common 6-histidines tag. Although the resulted recombinant his-tagged ALDH3A1 was expressed at remarkably higher rates, almost the whole amount of the recombinant protein was insoluble and trapped into the inclusion bodies (Figure 3A, Table 2). Unlike the case of MBP-fused expression, lowering the induction temperature at 25°C (Figure 3B) and at 18°C (Figure 3C) did not improve significantly the solubility of the recombinant protein (Table 2).

### Auto-induction Leads to Enhanced Expression Levels of his-tagged ALDH3A1 but Poor Solubility

In order to improve the solubility of the his-tagged recombinant ALDH3A1, we employed the auto-induction protocol described recently by Studier et al [21]. An important factor during heterologous protein expression is the need for a strict control of protein induction and the retention of cultures’ viability for as long as possible. The auto-induction protocol requires specialized culture media which in combination with high rates of aeration allows for a firm hold of the induction, an equilibrated pH and a subsequent elongated viability even at extremely high cell densities. Isopropyl β-D-1-thiogalactopyranoside (IPTG) induction is not applied in this case, as glucose, glycerol and lactose are all included in the media. Glycerol is used as an efficient carbon and energy source, which contributes to the growth of cells. On the other hand, as long as glucose is available in the media and usually until the late log phase, bacteria do not metabolize lactose. Near saturation, though, and when glucose is depleted, cells metabolize lactose to the inducer allo-lactose and the induction of the protein begins then manually with no extra addition. Due to the high viability of cultures, the yield of recombinant protein is extremely high.

**Figure 4:**
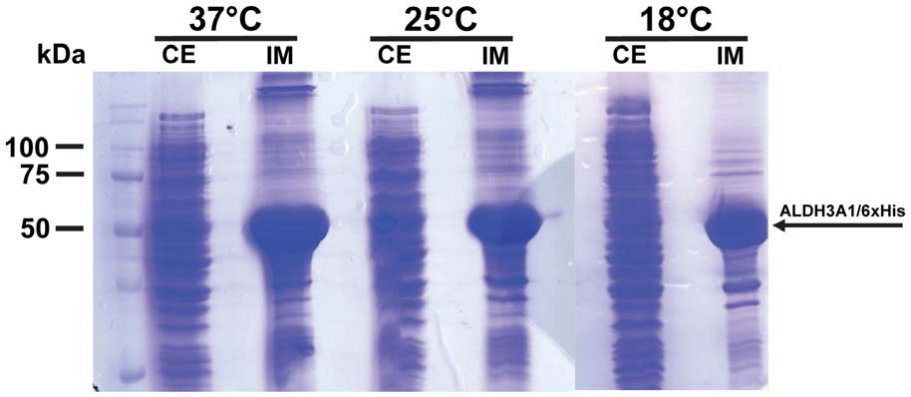
ALDH3A1 heterologous expression through autoinduction with the pET-26b(+) expression system. Samples were subjected to SDS-PAGE and stained with Coomassie blue. 37°C, 25°C and 18°C: different induction temperatures, CE: Crude extract of lysed cells 9 hours after inoculation, IM: Insoluble matter of lysed cells 9 hours after inoculation. The arrow indicates the position of ALDH3A1/6xHis protein.

In the case of ALDH3A1, while the expression of the protein was sufficiently rich under conditions of auto-induction, almost all of the produced ALDH3A1 was found in the inclusion bodies, as inactive aggregates (Figure 4). The soluble fraction of the expressed ALDH3A1 estimated to represent approximately 3% of the induced protein at 37°C (Table 2). Lowering the temperature from 37°C to 25°C and 18°C did not improve any further the solubility of the recombinant protein (Figure 4; Table 2).

### Molecular Chaperones’ Co-expression Facilitates the Production of Soluble Recombinant his-tagged ALDH3A1

Molecular chaperones play an important role in the conformation process of newly synthesized proteins. In the case of heterologous expressed proteins, which commonly exhibit solubility problems and misfolding, the presence of chaperones could become even more of a necessity. *E.coli* affords a variety of proteins that could be characterized as chaperones, and amongst them, the GroEL/GroES and the DnaK/DnaJ/GrpE are considered to be key groups. The above, along with the Trigger factor could be co-expressed with a heterologous protein and assist to its production in native and active forms. As these chaperone molecules are normally expressed at low levels in prokaryotic cells, heterologous over-expression of eukaryotic proteins with chaperones has been shown to improve the solubility of the overexpressed proteins in *E.coli* [24].

**Figure 5:**
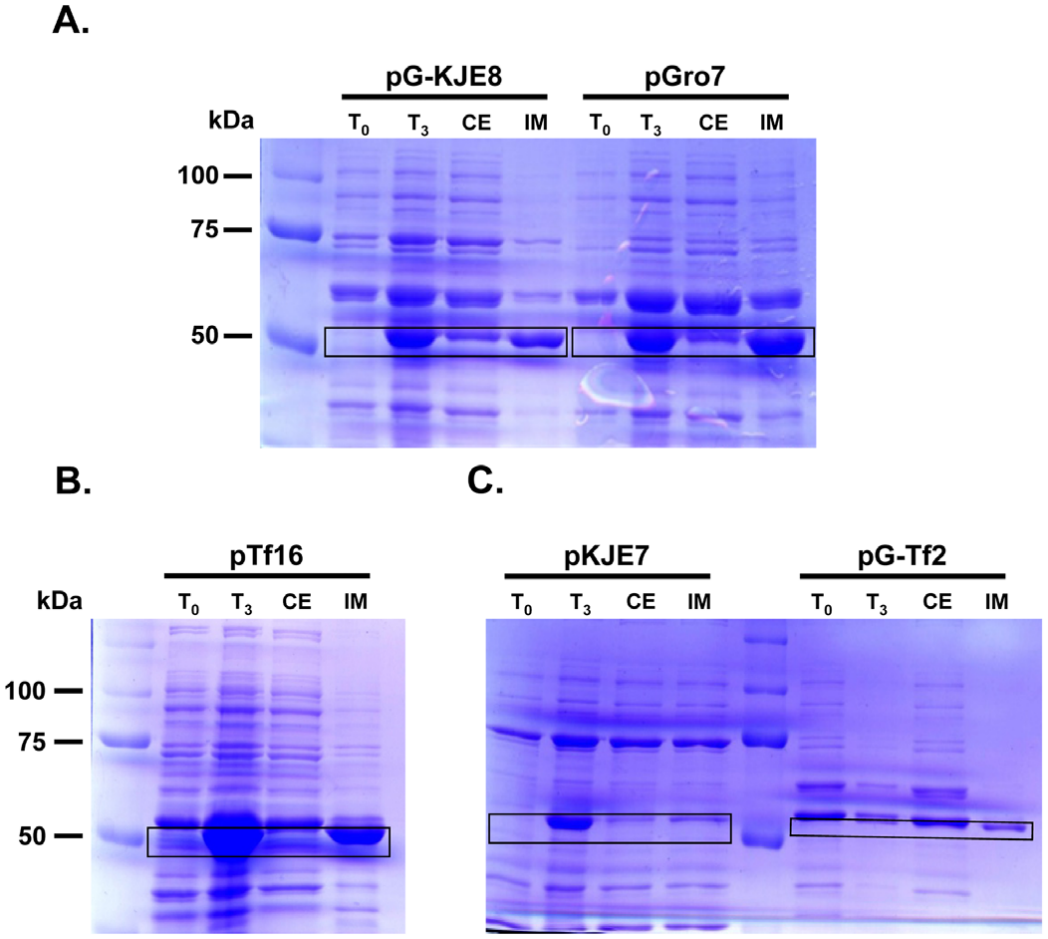
Co-expression of ALDH3A1 with molecular chaperones. (A) Co-expression of pG-KJE8/pGro7 plasmid’s chaperones, (B) Co-expression of pTf16 plasmid’s chaperones, (C) Co-expression of pKJE7/pG-Tf2 plasmid’s chaperones. Samples were subjected to SDS-PAGE and stained with Coomassie blue. T_0_: Total cell extract form bacterial culture prior of protein induction, T_3_: Total cell extract 3 hours after induction, CE: Crude extract of lysed cells 3 hours after induction, IM: Insoluble matter of lysed cells 3 hours after induction. The recombinant proteins expression is presented in the boxes.

In this study, we used different combinations of chaperones to enhance the solubility of the recombinant his-tagged ALDH3A1 in BL21(DE3) *E. coli* (Table 1). The solubility was improved in the cases of co-expression with the pG-KJE8 (approximately 20%), and pGro7 (approximately 12%) plasmids (Figure 5; Table 2). However, the expression levels of soluble his-tagged ALDH3A1 were significantly lower (3.7%) when the recombinant protein was co-expressed in the presence of pG-Tf2 plasmid (Figure 5C; Table 2). On the other hand, negligible enhancement in solubility was observed when his-tagged ALDH3A1 was co-expressed with pTf16 (approximately 6%) and pKJE7 (approximately 4%) (Figure 5B/C; Table 2). Our results demonstrate that the presence of chaperone complexes especially those contain GroES and GroEL (e.g. plasmids pGKJE8 and pGro7) increased the solubility of the recombinant protein. In the presence of *Tig* chaperone, the solubility of his-tagged was significantly impeded. Combination of GroES/GroEL and dnaK/DnaJ/GrpE chaperone groups exhibited the most profound effect on his-tagged ALDH3A1 solubility. Lower induction temperatures or addition of ethanol did not facilitate any further the protein’s solubility (data not shown).

### Purification of the Recombinant Human Fused ALDH3A1 Hybrids

Recombinant human fused ALDH3A1 hybrids were produced and purified from *E.coli* using the MBP-fused expression strategy (under low temperature conditions during protein induction) and the his-tagged ALDH3A1 expression strategy (under conditions of co-expressing the pG-KJE8) both of which produced soluble recombinant ALDH3A1 at sufficient levels.

**Figure 6:**
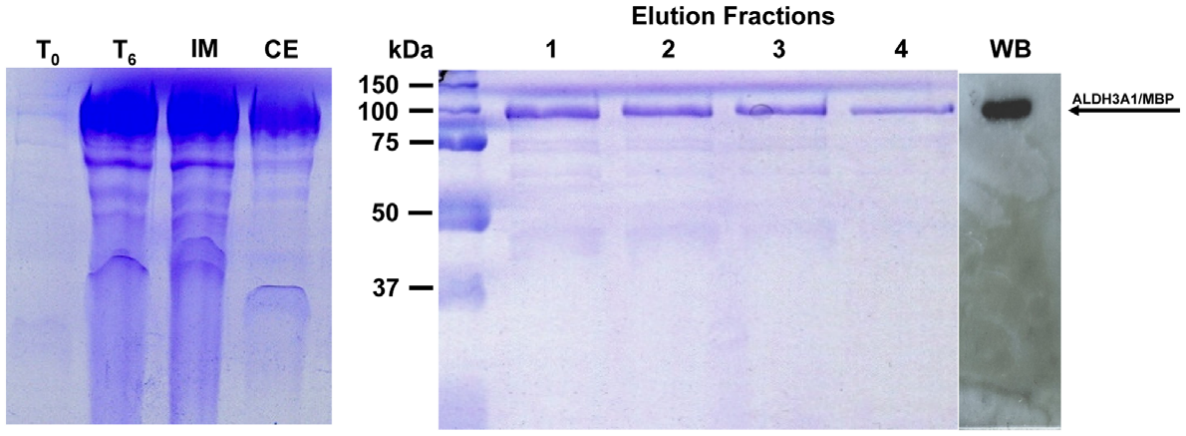
Protein expression and purification of recombinant MBP fused ALDH3A1. SDS-PAGE analysis at various stages of purification of recombinant MBP-fused ALDH3A1 using amylose resin chromatography (Coomassie blue staining). T_0_: Total cell extract form bacterial culture prior of protein induction, T_6_: Total cell extract 6 hours after IPTG induction, IM: Insoluble matter of lysed cells 6 hours after IPTG induction, CE: Crude extract of lysed cells 6 hours after IPTG induction, Elution fractions: purified recombinant ALDH3A1 eluted from amylose resin column. WB: western immunoblotting of purified recombinant ALDH3A1/MBP. The arrow indicates the position of the MBP-fused ALDH3A1 recombinant protein at approximately 92 kDa.

Purification of MBP-fused recombinant human ALDH3A1 was conducted with the use of affinity chromatography. Protein supernatant obtained from *E. coli* lysate was applied to amylose resin column in column buffer (see Methods). MBP-fused ALDH3A1 was then eluted from the column in the same buffer containing 10 mM maltose and appeared in the elution fractions (Figure 6). This convenient step of immobilized affinity chromatography resulted in purified recombinant MBP-fused ALDH3A1 of sufficient homogeneity. The final yield was approximately 5% from the initial 62.5 mg of crude protein and purification of the recombinant protein was 2.8-fold (Table 3).

**Figure 7:**
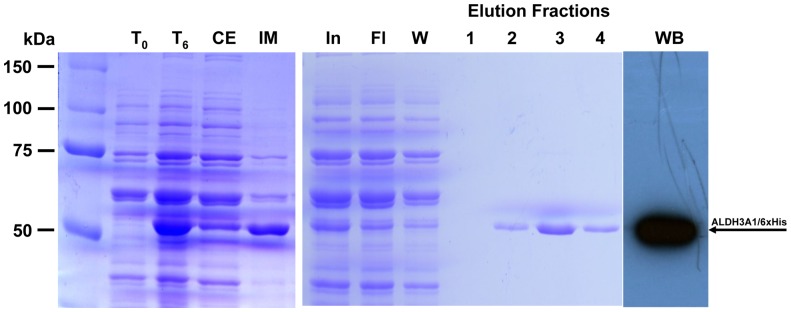
Protein expression and purification of recombinant his-tagged ALDH3A1. SDS-PAGE analysis at various stages of purification of recombinant his-fused ALDH3A1 using Ni-affinity chromatography (Coomassie blue staining). T_0_: Total cell extract form bacterial culture co-expressing pG-KJE8 along with ALDH3A1 prior to IPTG induction, T_6_: Total cell extract 6 hours after IPTG induction, CE: Crude extract of lysed cells 6 hours after IPTG induction, IM: Insoluble matter of lysed cells 6 hours after IPTG induction, In: Input of the column, Fl: flowthrough part, W: wash part, Elution fractions: purified recombinant protein eluted from Ni-NTA column. WB: western immunoblotting of purified recombinant ALDH3A1/6xHis. The arrow indicates the position of the recombinant his-tagged ALDH3A1 at approximately 51 kDa.

Isolation of his-tagged recombinant human ALDH3A1 was achieved by convenient one step affinity chromatography using nickel nitrilotriacetic (Ni-NTA) resin and elution in buffer containing 300 mM imidazole resulting in purification of his-tagged recombinant human ALDH3A1 to homogeneity (Figure 7). The final yield was approximately 7% from the initial 36 mg of crude protein and purification of the recombinan protein was 5.8-fold (Table 4).

**Table 4:** Purification of his-tagged recombinant human ALDH3A1 from *E. coli.*

Purification Steps	Total Protein (mg)	Yield (%)	Specific Activity^b^ (mU/mg protein)	Purification (fold)
Crude supernatant^a^	36	100	200	1
Ni-NTA column	2.43	6.75	1150	5.75

a The starting material was 250 ml of crude *E. coli* supernatant.

b One milliunit (mU) of activity was defined as the amount of activity that oxidized of 1 nmol of NADPH/min at 25°C. Representative results of three different isolation procedures.

## Discussion

Previous attempts to express active recombinant human ALDH3A1 in *E. coli* have failed primarily because of low solubility, yield and insufficient purity issues. In this study, we compared different *E. coli* fusion expression strategies (the MBP- and the 6-his-tagged expression) under various conditions intending to increase their efficiency for producing soluble recombinant ALDH3A1. We have shown that the MBP-tagged expression in combination with lower- temperature culture conditions resulted in active soluble recombinant ALDH3A1. Expression of the fused his-tagged ALDH3A1 protein resulted in poor solubility and neither lowering temperature culture conditions nor auto-induction conditions improved solubility. Furthermore, higher yield of soluble, fully active native form of his-tagged ALDH3A1 was facilitated through the co-expression of the two groups of *E.coli*’s molecular chaperones GroES/GroEL and DnaK/DnaJ/GrpE. Convenient one-step immobilized affinity chromatography methods were utilized to purify the fused ALDH3A1 hybrids to sufficient homogeneity. To our knowledge, this is only the second time that recombinant techniques have been used to produce human crystallin ALDH3A1. We have previously utilized the baculovirus expression system to produce recombinant human ALDH3A1 in order to overcome the insolubility problems occurred at our initial attempts to express the protein in *E. coli*. The baculovirus infection system in Sf9 cells in combination with 5′ AMP sepharose chromatography resulted to extremely high yield of recombinant ALDH3A1 with sufficient solubility [5]. However, while it is true that the baculovirus expression systems allow for high yields of structurally and functionally foreign proteins in insect cells, their high cost, complexity and requirement of specific equipment and trained personnel pose limitations thus providing reasoning for the development of alternative recombinant expression strategies.

Insolubility is the number one issue of recombinant proteins expressed in *E. coli*. The reason for this is believed to be their non-native, aggregation-prone conformation and their subsequent expression as inactive forms in the inclusion bodies. Important parameters include the interactions between the hydrophobic patches of newly synthesized unfolded polypeptides, which are influenced by the rate of protein synthesis along with the cellular microenvironment of expression. Therefore, factors considered important during protein synthesis are usually related with the expression vector used, the induction parameters and the cultivation conditions [17].

The selection of the appropriate tag for the fusion of the heterologous protein usually depends on the desired method of chromatography and the experimental needs of the protein’s utilization. On the other hand, protein solubility depends on the type of the fusion tag to be used. Tags known for their contribution to solubility are the MBP, the NusA and the GST (glutathione S-transferase) [19]. MBP**,** in particular, is considered to be one the most effective and has been widely used. Although, MBP appears to facilitate the correct formation of disulfide bonds in the newly synthesized proteins, the exact mechanism through which it improves the solubility of the target heterologous proteins remains unknown [25]. Indeed, in our case, the expression of MBP-fused ALDH3A1 in combination with lower induction temperature in *E. coli* resulted in much better solubility compared to his-tagged ALDH3A1 expression under all different strategies tested.

While the establishment of a highly productive system with strict control elements is a well known and crucial issue, another prominent, but often underestimated factor is the need for high cell density cultures with enhanced viability [16]. Even though *E. coli* cultures are easy to be cultivated, the limited sources of oxygen and nutrition elements as well as the increased energy needs under conditions of protein induction, lead to stress and inadequately operated metabolism. This is the reason why the induced protein expression occurs during the exponential rather than the stationary phase, but even in this case, problems could also occur and the yield of production could be significantly restrained [13]. Auto-induction, a technique introduced by Studier et al [21], was designed so as to solve the above restrictions. Contrary to the classic method, protein expression is induced near saturation phase, when cultures have a high cell density, by lactose supplementation in the media instead of IPTG. Induction prior to saturation is prevented by glucose. The specially formulated media required along with proper aerization conditions are responsible for balancing the pH of the cell cultures and the growth of cells to extremely high densities without loss of viability. Bibliography is full of variable examples of correctly expressed proteins using the auto-induction method. The tumor necrosis factor family member APRIL [26], long repetitive resilin-based proteins [27], the psychrophilic TAB5 alkaline phosphatase [28], an Asc-1 homologue [29], the human epoxide hydrolase [30], the holotoxin Stx2 [31], and globin chains from Arenicola marina [32] are just a few of the recombinant produced proteins to name. In the case of recombinant ALDH3A1 (his-tagged ALDH3A1) however, the employment of auto-induction method resulted in higher protein expression levels but unfortunately it did not improve the protein’s solubility.

Considering cultivation conditions, several different strategies have been demonstrated, in everyday laboratory practice, for the enhancement of protein solubility. The most commonly and easily tested one is with no doubt the low-temperature culture which was proved to be miraculous in a variety of cases [20], [33] including ours as well by enhancing the solubility of the MBP-fused ALDH3A1. On the other hand, temperature is known to correlate with the production of active protein through a variety of mechanisms. Hydrophobic interactions, the basic driving force of inclusion bodies formation depend on temperature. Furthermore, (i) the temperature-dependent expression of molecular chaperones, (ii) the reduction of protein synthesis rate, (iii) the different folding kinetics and (iv) the lower activity of specific proteases [17], [18] can also contribute to the enhanced yield of active recombinant proteins.

Co-expressing the recombinant protein with chaperones has been exploited as the most effective way and a quality control system to increase the solubility of recombinant proteins in *E. coli* [34]–[37]. Molecular chaperones facilitate the correct conformation of newly synthesized proteins and contribute in the retention of their native folding. Among them, DnaK/DnaJ/GrpE along with GroES/GroEL are the most commonly used systems for the expression of soluble proteins [22], [38]. Basically, they are ATP-dependent folding chaperones which induce the partial unfolding and re-folding of non-native proteins [17]. Trigger factor, on the other hand, associates with the synthesized proteins as soon as they leave ribosome and through its interaction with their exposed hydrophobic patches averts their subsequent aggregation [14], [38]. Co-expression of molecular chaperones resulted in enhanced solubility and production of recombinant rice plant catalase A [39] active ribonuclease inhibitor [40], human scramblase 1 [41] and zeta-crystallin [42]. Solubility of his-tagged ALDH3A1 was significantly improved under conditions of co-expressing the pG-KJE8) suggesting that dnaK/dnaJ/grpE and groES/groEL are the essential chaperones for the correct folding of recombinant human ALDH3A1 (his-tagged ALDH3A1) when over-expressed in *E. coli*.

In summary, soluble MBP-fused and his-tagged recombinant human ALDH3A1 proteins have been successfully expressed in *E. coli* and purified to homogeneity. Both fusion proteins retained their biological activity and so can be used directly without removing the fusion tags. The methods described in this study permit the production of substantial amounts of the recombinant human ALDH3A1 for conducting functional studies on the biological role of this interesting crystallin, which exists in high concentrations in the cornea of certain mammalian species.
